# Anisotropy of radiation-induced defects in Yb-implanted β-Ga_2_O_3_

**DOI:** 10.1038/s41598-024-75187-6

**Published:** 2024-10-22

**Authors:** Renata Ratajczak, Mahwish Sarwar, Damian Kalita, Przemysław Jozwik, Cyprian Mieszczynski, Joanna Matulewicz, Magdalena Wilczopolska, Wojciech Wozniak, Ulrich Kentsch, René Heller, Elzbieta Guziewicz

**Affiliations:** 1 National Centre for Nuclear Research, ul. Soltana 7, Otwock, 05-400 Poland; 2 Inst. of Physics, Polish Acad. of Sciences, Aleja Lotnikow 32/46, Warsaw, PL-02668 Poland; 3 Helmholtz-Zentrum Dresden-Rossendorf, Bautzner Landstrasse 400, Dresden, D-01328 Germany

**Keywords:** Wide bandgap semiconductors, Gallium oxide, Ion implantation, Radiation defects, Rutherford Backscattering Spectrometry, Channeling, Condensed-matter physics, Materials for devices

## Abstract

RE-doped β-Ga_2_O_3_ seems attractive for future high-power LEDs operating in high irradiation environments. In this work, we pay special attention to the issue of radiation-induced defect anisotropy in β-Ga_2_O_3_, which is crucial for device manufacturing. Using the RBS/c technique, we have carefully studied the structural changes caused by implantation and post-implantation annealing in two of the most commonly used crystallographic orientations of β-Ga_2_O_3_, namely the (-201) and (010). The analysis was supported by advanced computer simulations using the *McChasy* code. Our studies reveal a strong dependence of the structural damage induced by Yb-ion implantation on the crystal orientation, with a significantly higher level of extended defects observed in the (-201) direction than for the (010). In contrast, the concentration and behavior of simple defects seem similar for both oriented crystals, although their evolution suggests the co-existence of two different types of defects in the implanted zone with their different sensitivity to both, radiation and annealing. It has also been found that Yb ions mostly occupy the interstitial positions in β-Ga_2_O_3_ crystals that remain unchanged after annealing. The location is independent of the crystal orientations. We believe that these studies noticeably extend the knowledge of the radiation-induced defect structure, because they dispel doubts about the differences in the damage level depending on crystal orientation, and are important for further practical applications.

**Supplementary Information:**

The online version contains supplementary material available at 10.1038/s41598-024-75187-6.

## Introduction

Nowadays, materials science is driven by new technologies that aim to improve existing materials and replace them with cheaper and more efficient equivalents. For several decades, semiconductor compounds have fit in perfectly with this trend, and are irreplaceable for optoelectronic applications such as lasers, displays, or light-emitting diodes (LEDs), where silicon cannot be used due to its indirect and not very wide bandgap (WBG).

Gallium sesquioxide in its thermodynamically stable beta phase (β-Ga_2_O_3_) is a transparent conductive material, which belongs to the group of WBG semiconductors (with energy band gap E_g_ of ~ 4.8 eV). The research focusing on this material started many decades ago, but interest in it has recently been revived due to its potential applications in many high-tech opto- and microelectronic devices. β-Ga_2_O_3_ also possesses other unique properties such as a combination of intrinsically high electron mobility, a high electrical breakdown field (~ 8 MV/cm)^1,2^, and the ability to be efficiently donor-doped^3,4^, which makes it a promising material enabling efficient distribution, use, and conversion of electrical energy. Its bandgap is wider than in currently leading WBG semiconductors like ZnO and GaN (E_g_ ~3.4 eV and ~ 3.2 eV, respectively) causing it to be solar-blind and fulfill optical transparency requirements significantly better^5^. It also has a fairly high refractive index and seems to be an efficient luminescent host material for a number of ions, which is why this oxide is of interest for waveguiding applications or for color displays^6^. Moreover, a wider bandgap is the reason for the increased thermal, radiation, and chemical robustness, which makes β-Ga_2_O_3_ less prone to aging and ideal for radiation-intense environment applications or military systems^7^.

The characteristic near band-edge light emission from β-Ga_2_O_3_ is located in the ultraviolet (UV) spectral range^8–10^. Nevertheless, doping with Rare Earth (RE) atoms can modify the optical response of this material and tune the optical emission into the visible region^11^. Unfortunately, doping of β-Ga_2_O_3_ with RE during the growth process is challenging due to the low solubility limits in the beta phase, which often leads to segregation or secondary phases^12–14^. An attractive, alternative method to doping, commonly used in electronics, is ion implantation, where the dopant concentration and depth profile can be precisely controlled by selecting the appropriate ion fluence and energy^15^. Moreover, the implantation is a strongly non-equilibrium process that allows the introduction of any kind of atoms into solids with concentrations well above the solid solubility limits. Despite many advantages, an important limitation of the implantation technique is the buildup of lattice disorder due to the ballistic nature of the process, causing a change in the electronic properties of β-Ga_2_O_3_ and decreasing its optical efficiency^16,17^. Fortunately, the implantation-induced damage process can be reversible and the crystal lattice can be recovered, e.g. by thermal annealing. However, it has already been shown that it is possible only below a certain critical fluence and using optimal conditions of the annealing process^18^. Hence, systematic studies of radiation-induced defects in RE-implanted β-Ga_2_O_3_ are desperately needed to understand the performance of such systems. It is essential to establish defect transformation thresholds, as well as the influence of the annealing on the formed defects and the level of structure recovery. Therefore, for future applications, the identification of ion-implantation-induced defects is crucial to eliminate them or use them in the so-called “defect engineering”.

Although a few papers on the defect structures created in β-Ga_2_O_3_ bombarded with heavy ions could be found in the literature, these reports are not fully consistent^19–25^. This is mostly because the radiation-induced defect accumulation process in β-Ga_2_O_3_, as well as the types of created defects, seem to depend strongly on many factors like quality, and manufacturing method of the target material, implantation conditions as well as the physicochemical properties of the dopant ions implanted into the crystal lattice^26–31^.

In the present work, we pay particular attention to the issue of radiation-induced defects anisotropy in β-Ga_2_O_3_. Using Rutherford Backscattering Spectrometry in the channeling mode (RBS/c), we have carefully investigated the structural changes in two crystal orientations most commonly applied crystallographic orientations, namely, the (-201) and (010), that were implanted with three different fluences of 1 × 10^13^, 1 × 10^14^, and 1 × 10^15^ Yb ions/cm^2^. According to the previous studies^30^ such fluences cause the damage near the defect transformation thresholds. The present studies are supported by the advanced Monte Carlo simulation code called *McChasy-1*, used for the quantitative analyses of depth defect profiles^32,33^. We have found a strong dependence of the structural damage induced by the Yb-ion implantation on the crystal orientation, with a significantly higher level of extended defects, like dislocations (or other defects resulting in channel bending), observed for (-201)-oriented β-Ga_2_O_3_ than that for (010)-oriented ones. In contrast, the concentration and behavior of simple defects seem similar for both crystal orientations. However, it is worth noting that the evolution of RDA-type defects is quite complicated and suggests the co-existence of two different types of simple defects created in the implanted zone, which have different sensitivities to both radiation and annealing. The studies shed new light on the perception of the post-implantation damage issue in β-Ga_2_O_3_, allowing the identification of the nature of the formed defects and their concentration in dependence on crystal orientation. We are truly convinced that our findings significantly extend current knowledge of the radiation-induced defect structure and will be important for potential future applications.

## Results and discussion

**Figure 1:**
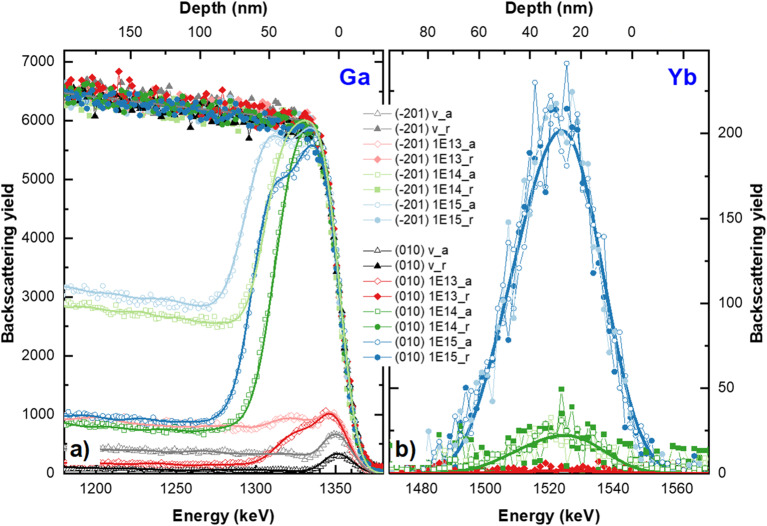
Random (solid symbols) and aligned (open symbols) RBS experimental spectra obtained for (-201) and (010) oriented β-Ga_2_O_3_ single crystals virgin and implanted with different fluences of Yb ions. Signals coming from Ga are shown in (**a**) and Yb in (**b**), respectively. Solid lines are results of MC simulations made with the *McChasy-1* code.

Typical RBS/c spectra obtained for β-Ga_2_O_3_ implanted with RE ions show separated signals coming from He-ions backscattered from Ga and RE atoms, due to their mass difference (see Fig. 1). Well-distinguishable signals mean that we are able to simultaneously monitor crystal lattice damage evolution based on the Ga signal (lower energy part of RBS spectra: 1100–1400 keV), and RE behavior (higher energy part: 1450–1600 keV), which is the advantage of the RBS/c technique in such analyses. The energy value that corresponds to the He ions backscattered from the Ga surface atoms is 1353 keV. The Ga-signal spreads towards the lower energy regime as a result of backscattering occurring deeper in the lattice. Due to the low cross-section on the backscattering He ions on the light atoms of the matrix, the signal coming from oxygen atoms (O), spreading from 616 keV, is hardly visible in the spectra and, additionally overlaps with the Ga signal at low energies. Thus, the O signal is not suitable for analysis and is not shown in Fig. 1 for clarity.

**Figure 2:**
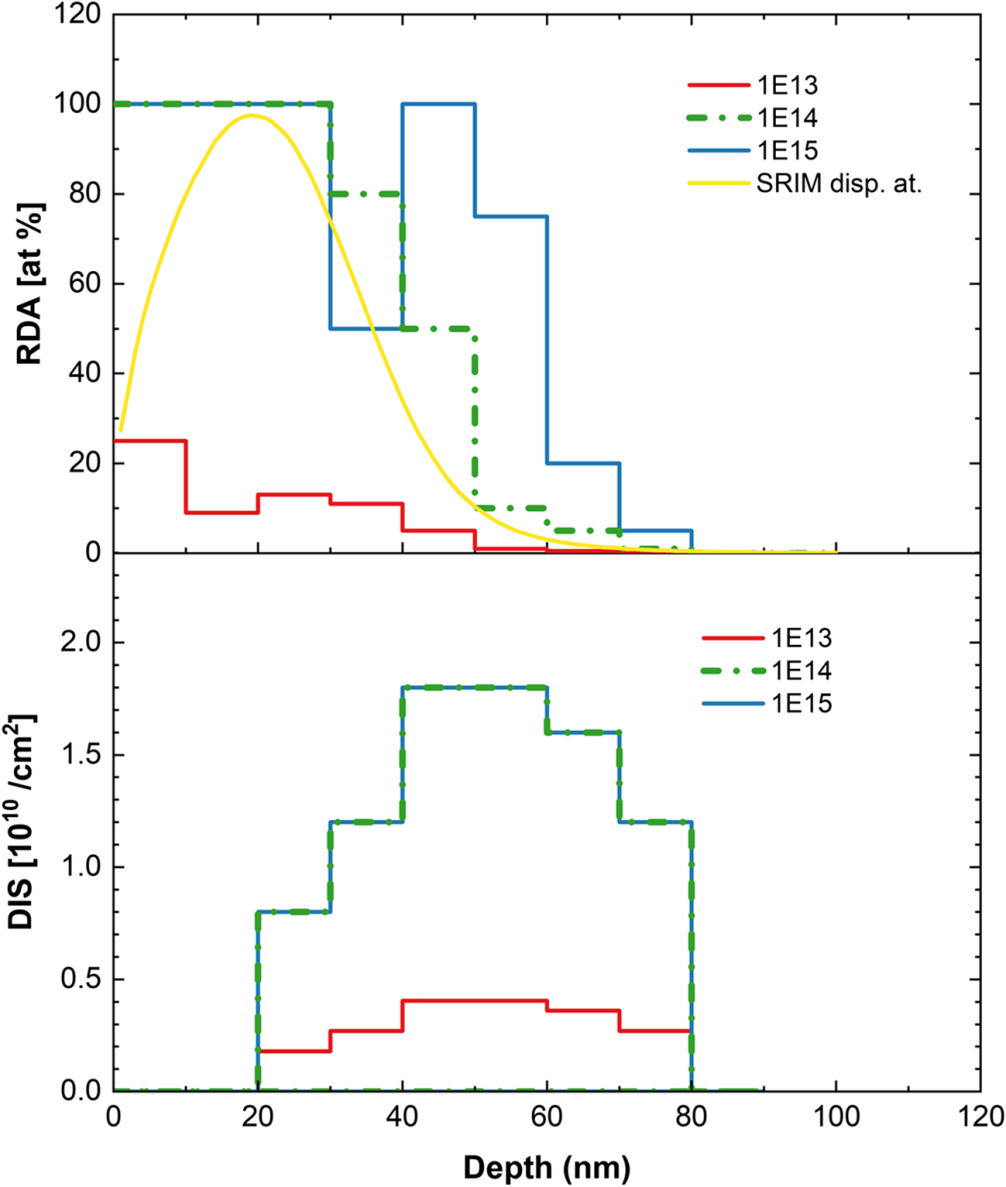
Distribution of simple (RDA) and extended (DIS) defects obtained by *McChasy-1* simulations for (010) β-Ga_2_O_3_ implanted with Yb-ions of different fluences. The yellow line shows displaced atom distribution predicted by SRIM.

The Yb signals becomes visible only above the fluence of 1 × 10^14^ cm^−2^ (see Fig. 1(b)). For all the fluences, which the Yb signal is visible, the shape of the Yb signal recorded in the channeling mode is similar to the corresponding measurement in the random mode. It means that the Yb ions occupy mainly the interstitial positions in the crystal lattice of β-Ga_2_O_3_ after the implantation. As can be seen, these crystal lattice-site positions of Yb atoms are the same for both oriented crystals. In contrast, looking at the Ga part of the spectra (Fig. 1(a)), several important differences in RBS-aligned spectra can be observed. Firstly, the considerably lower dechanneling level for the (010)- oriented β-Ga_2_O_3_ crystals compared to the (-201) is seen. These differences already appear for undoped β-Ga_2_O_3_ crystals showing that differently oriented crystals have different sensitivities to backscattering due to different shapes and sizes of the crystal channels^34^. The χ_min_ parameter is a numerical evaluation of this observation^35^. Its value, calculated as the ratio of the aligned spectrum to the random one in the same energy region is 5.7% and 1.3% for the (-201) and (010)- oriented β-Ga_2_O_3_, respectively. Usually, the χ_min_ parameter is a measure of the crystalline quality. Nevertheless, in this case, it demonstrates the aforementioned different sensitivity of the analyzing ions used in RBS/c techniques to the backscattering, since the HRXRD results presented in our other publication^18^ show the sharp signal coming from the symmetric (-402) reflection of undoped (-201) oriented β-Ga_2_O_3_ crystals with full width at half maximum (FWHM) as 0.012 (°), calculated from the rocking curve, which provides evidence of the good quality of the initial crystals used in the experiment. Secondly, for the implanted samples an increase in the so-called damage peaks with increasing ion fluence is observed, which reflects enhanced radiation-induced crystal disorder in the implanted zone. In the RBS-aligned spectra, the damage peaks occur in the energy range of ~ 1300–1360 keV, which corresponds to 0–80 nm in the depth range. According to theoretical SRIM calculations^36^, the damaged region should be narrower (see Fig. 2). This becomes particularly visible when the higher fluence is used, indicating the complicated kinetics of defect accumulation in the tested material.

**Figure 3:**
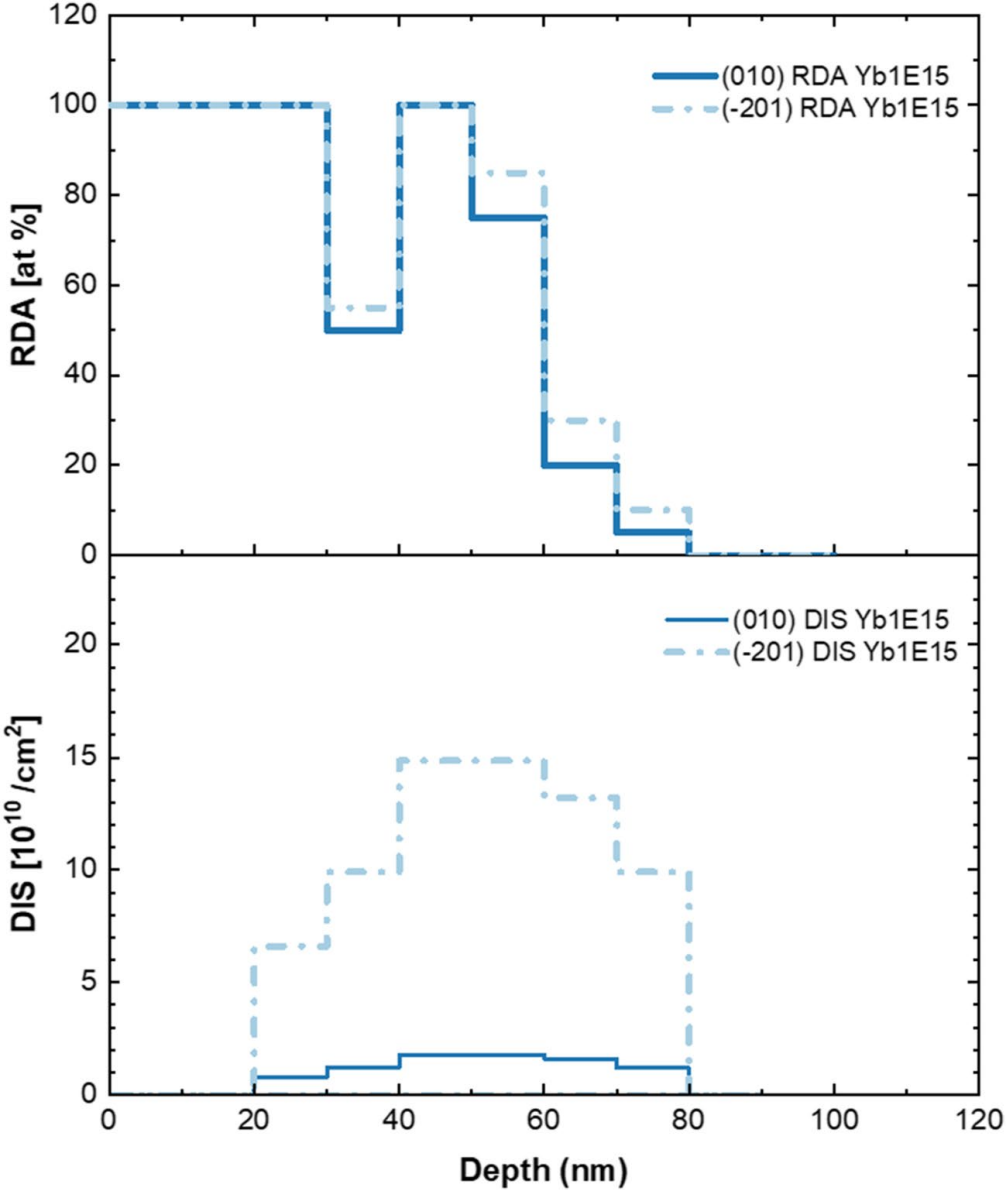
Distribution of simple (RDA) and extended (DIS) defects obtained by *McChasy-1* simulations for the (010) and (-201) oriented β-Ga_2_O_3_ implanted with Yb-ions fluence of 1 × 10^15^ /cm^2^.

The solid lines in Fig. 1 represent the results of the simulations performed by the *McChasy-1* program^32,33^, which was used for a detailed analysis of the defect structures. By performing such simulations, it is possible to extract information about the defect depth distributions for two different types of defects independently: simple and extended. Simple defects are modeled in the code as randomly displaced atoms (RDA), which are considered as a total value of defects causing the direct backscattering of the analyzing ions. In turn, extended refer to those that cause the bending of crystal channels like edge dislocations (DIS) and hence interact with the beam by contributing to the dechanneling effect rather than to the direct backscattering. The defect profiles obtained by *McChasy-1* are shown in Figs. 2 and 3.

The defect depth profiles obtained for the (010)- oriented β-Ga_2_O_3_ implanted with different Yb ion fluences, shown in Fig. 2, reflect the observations that for two lower Yb fluences, i.e. 1 × 10^13^ and 1 × 10^14^ at/cm^2^, the RDA damage peak is single, but for the higher fluence, i.e. 1 × 10^15^ at/cm^2^, it becomes bimodal. For both higher fluences, the concentration of the RDA-type defects reaches saturation at the level of 100% (random level), and the concentration of the DIS-type defects is constant in this fluence range. This indicates that the damage behavior progress is quite complicated, but develops similarly for both investigated orientations (see Fig. 4 in Ref^30^ and Figure S1 in Supplementary Material (SM)).

**Figure 4:**
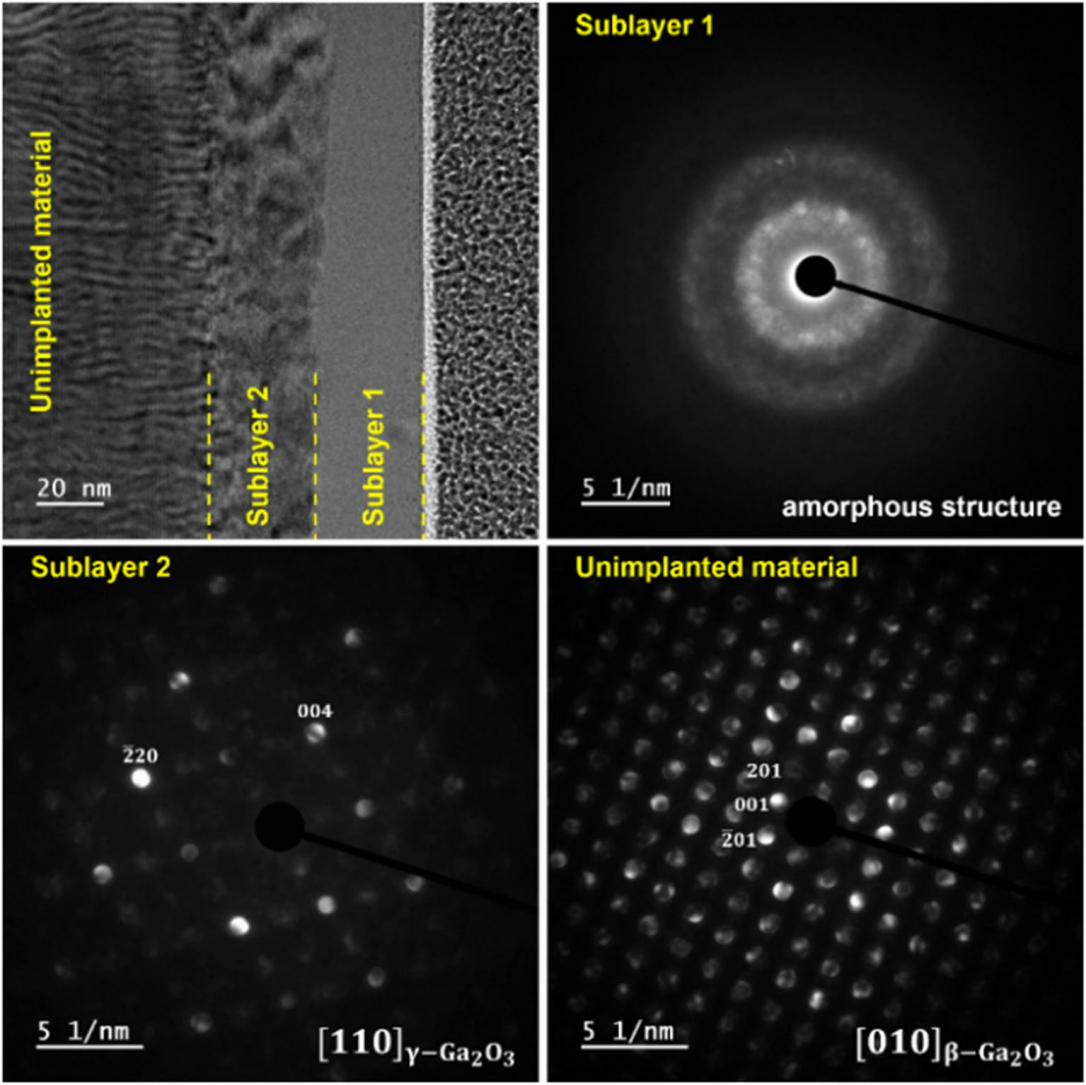
The BF-TEM image of the β-Ga_2_O_3_ implanted with 1 × 1015 Yb ions/cm2 with the corresponding SAED patterns from the selected areas.

Our studies, which have already been presented in Ref^30^. revealed four stages of damage accumulation in Yb-bombarded (-201) β-Ga_2_O_3_. The lowest Yb fluences used in this work, i.e., 1 × 10^13^ at/cm^2^, corresponds to the fluence region reffering to stage I, where the low concentration of defects is due to so-called dynamic annealing, that involves migration, and recombination of mobile point defects^15^. It is also the critical fluence, after which the first defect structure transformation in the crystal has just begun leading to the formation of dislocation tangles, and two different RDA–types of defects. It was considered, that the simultaneous development of these two different RDA types of defects in the structure caused the stress, which cumulatively becomes a starting point for migration (or pushing) one of the RDA-type defects into the depth and the growth and spread of another RDA-type defect near the surface. This occurs around the next fluence used in this work, i.e., 1 × 10^14^ at/cm^2^. Above 1 × 10^14^ at/cm^2^ observed in RBS/c spectra the damage peak became bimodal, and the simulation by McChasy clearly shows two separate damage zones. Finally, for the highest fluences used, i.e., 1 × 10^15^ at/cm^2^, the migration and evolution processes of RDA-type defects seem to be finished. However, the next transformation of the defect structure starts to become visible.

The TEM studies presented in Fig. 4 confirm the existence of two separate damage zones in the (-201) β-Ga_2_O_3_ crystal implanted with a higher fluence of Yb, designated as sublayers 1 and 2, respectively. Sublayer 1 is near the surface (0–30 nm), while sublayer 2 is localized deeper (30–70 nm), which is consistent with the RBS/c results. The corresponding Selective Area Electron Diffraction (SAED) patterns taken from the area of selected layers recognized sublayer 1 as an amorphous phase, while the deeper sublayer was identified as the γ-phase of Ga_2_O_3_. The existence of an implantation-induced γ-phase is also clearly demonstrated by HRXRD see Ref. ^30^.

**Figure 5:**
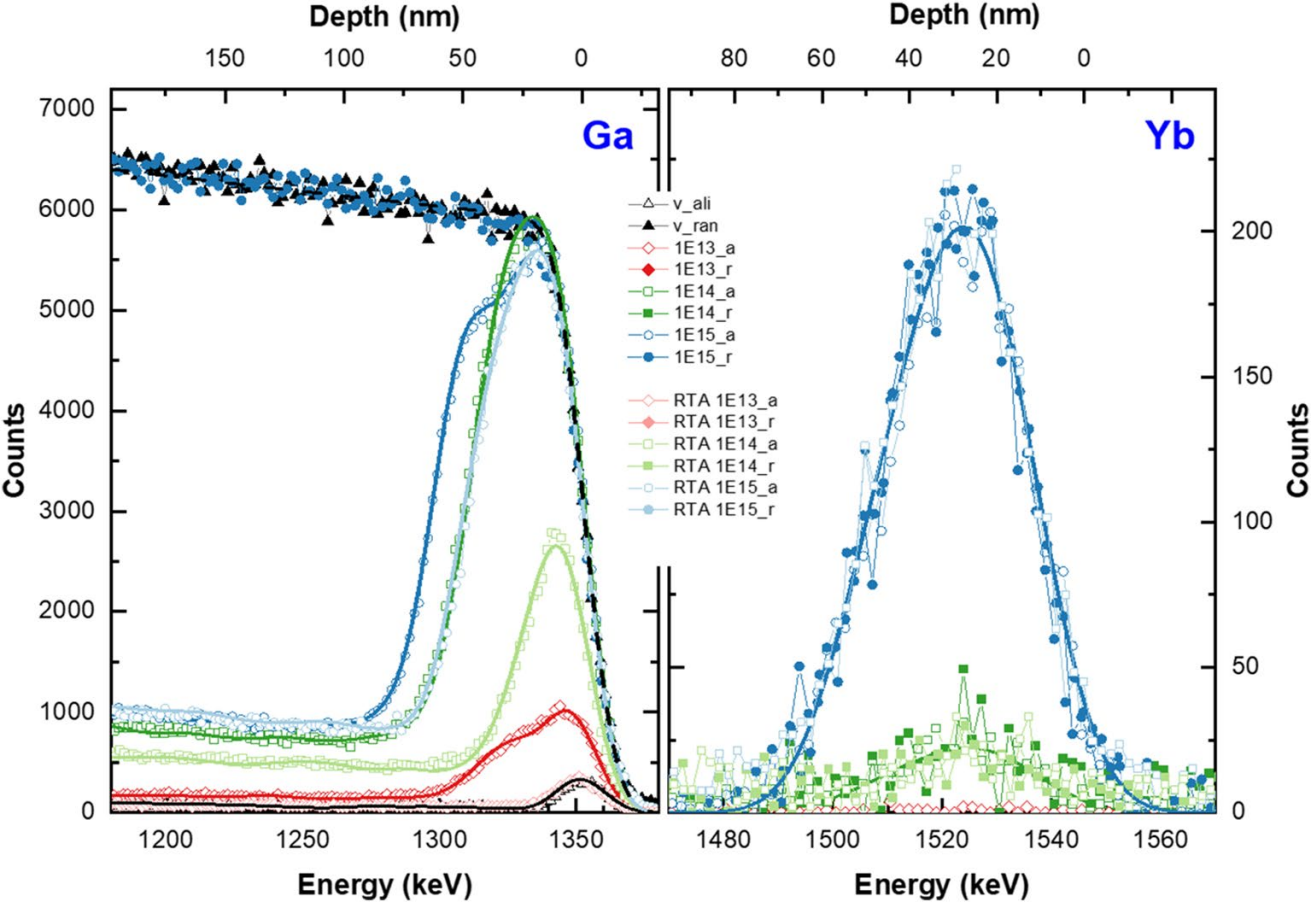
Random (solid symbols) and aligned (open symbols) RBS experimental spectra obtained for (010) oriented β-Ga_2_O_3_ single crystals implanted with different fluences of Yb ions and annealed in O_2_ at 800^o^C for 10 min. Solid lines are results of MC simulations made with the McChasy-1 code.

Although the defects’ behavior appears to progress in the same manner for the (010) and (-201)- oriented β-Ga_2_O_3_ crystals, the comparison of the defect depth distributions obtained for differently oriented implanted crystals reveals one significant difference, as shown in Fig. 3. Namely, it can be clearly seen that the concentration of RDA-type defects is similar in both oriented crystals, while the concentration of extended defects is significantly higher for the (-201)- oriented β-Ga_2_O_3_ crystals than that for (010)- oriented (Table 1). The observed differences in the dechanneling level for the implanted crystals are relatively much higher than in the case of the pristine samples of different orientations. This means that dislocations (or dislocation loops) are formed much easier along the (-201) direction indicating the preferable orientation for their formation.

**Table 1:** The maximum values of defect concentrations.

Fluence [at/cm^2^]	(010) β-Ga_2_O_3_		(-201) β-Ga_2_O_3_	
	RDA [at %]	DIS [10^10^ /cm^2^]	RDA [at %]	DIS [10^10^ /cm^2^]
1 × 10^13^	13	0.41	11	7.88
1 × 10^14^	100	1.80	100	14.85
1 × 10^15^	100	1.80	100	15.30

**Figure 6:**
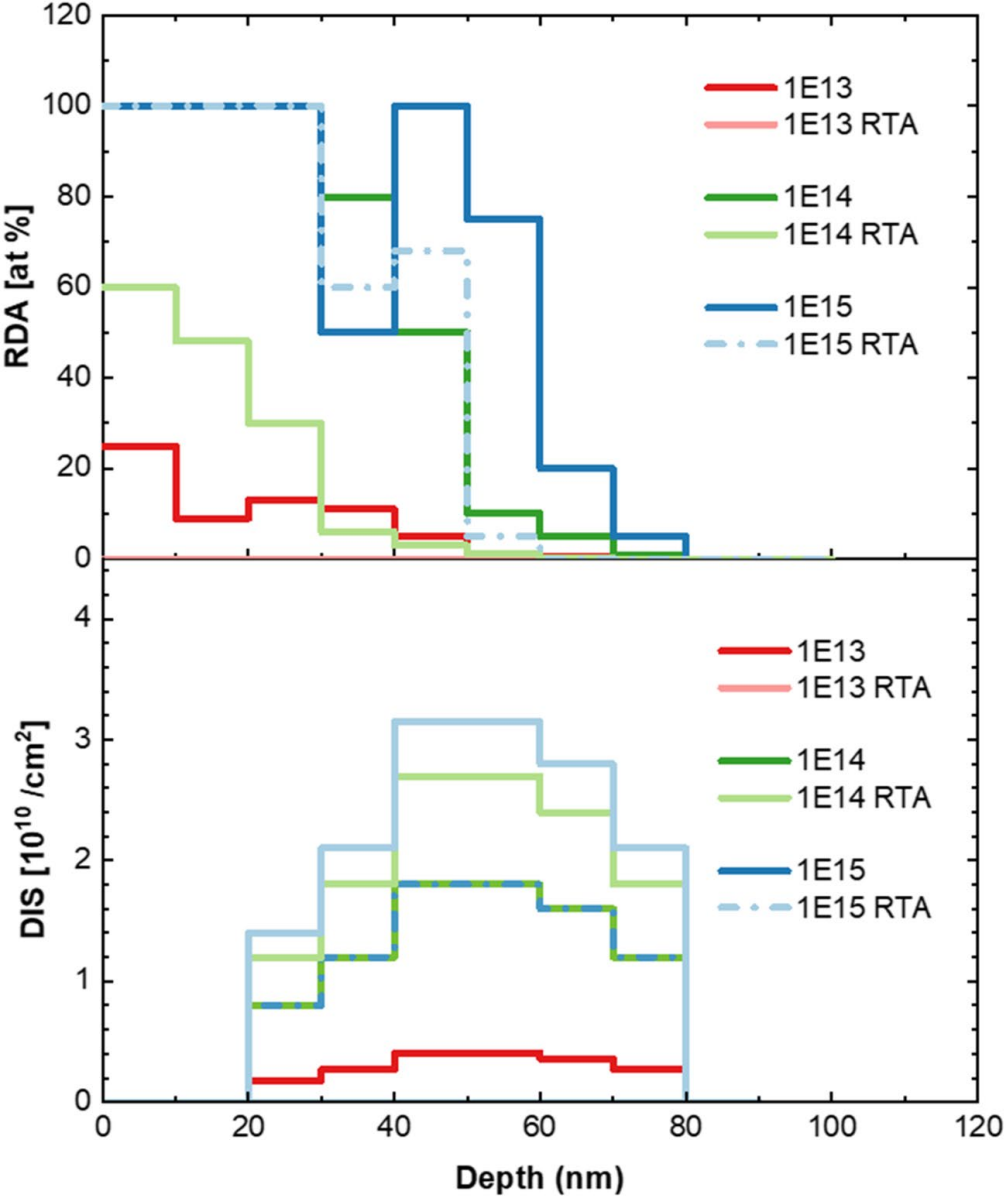
Distribution of simple (RDA) and extended (DIS) defects obtained by *McChasy-1* simulations for (010)- oriented β-Ga_2_O_3_ single crystals implanted with different fluences of Yb ions and annealed in O_2_ at 800^o^C for 10 min.

The results of the thermal treatment of the samples performed with the RTA system at 800 °C, for 10 min. in an oxygen atmosphere are shown in Figs. 5 and 6. Such annealing conditions were established as optimal for optical applications of β-Ga_2_O_3_ implanted with rare earth ions^18^. Figure 5 shows the set of experimental and simulated RBS spectra obtained for implanted and subsequently annealed (010)- oriented β-Ga_2_O_3_ crystals, respectively, while Fig. 6 illustrates the corresponding defect distributions obtained from the MC simulations using the *McChasy-1* code. Looking at the Yb signals in Fig. 5, it can be seen that there is no change in the position of the RE ions regarding the lattice site or within the depth after annealing for both oriented crystals. The Ga-signal part of the spectra indicates that the crystal damaged at the the fluence of 1 × 10^13^ at/cm^2^ could be fully recovered by applying the annealing under the described conditions, while in the case of higher fluences the crystal recovery is only partial.

The same effect is also observed in the case of the (-201)- oriented crystals (see Figures S2 and S3 in the SM). Other interesting observations can be seen in Fig. 5 for the highest fluence, i.e., 1 × 10^15^ /cm^2^. Recall that at this fluence, the damaged zones are well separated after implantation for both (-201) and (010)-oriented β-Ga_2_O_3_. In this case, the annealing strongly affects the thickness of the damaged zone, while the dechanneling level of the spectra has changed only slightly.

The reasons for this become clear by looking at the details in defect distributions shown in Fig. 6 (for the corresponding figure for the (-201)-oriented β-Ga_2_O_3_, see Figure S4 in SM and ref^30^. ). As can be seen, from the *McChasy* simulation results, the deeper RDA-type defects disappear after the annealing procedure, while the RDA damage zone closer to the surface seems to remain unchanged. Our HRXRD results presented in Ref^30^. clearly showed that for the Yb-implanted (-201) β-Ga_2_O_3_ crystals, the radiation-induced γ-phase disappeared after annealing. However, the additional peak associated with strain can be observed, suggesting that the thermal recovered β-phase remains partially defective. For ion-implanted (010) oriented β-Ga_2_O_3_ crystals, such β-γ-β phase transitions have been widely observed by other authors^24,28,29,31^.

Interestingly, *McChasy* simulations also show that the concentration of DIS -type of defect increases after the annealing. A similar effect of the increase in the extended defect amount after annealing have been already reported in the literature, and explained as the formation of entangled dislocations^37^. In our case, the Yb-O clustering can also be expected^38^. Furthermore, the newest works suggest that the radiation-induced γ- phase is a disordered version of the β-Ga_2_O_3_, where Ga ions of β-Ga_2_O_3_-lattice are moved towards interstitial positions^39,40^. Hence, the radiation-induced γ- phase transition makes a lot of free vacancies, which become mobile during the annealing and could agglomerate. Such nano-sized voids created during annealing have been identified in β-Ga_2_O_3_ implanted with Sn and Si ions^27,39^. Both dislocations and voids bend the crystal channels and could be a justifiable reason for the increase in DIS-type defects observed by RBS/c after annealing. On the other hand, the recent work also suggests another reason, which could be a rational explanation for this observation. Namely, Azarov et al. observed that the γ-to-β transformation after annealing at temperatures above 700 °C resulted in a differently oriented β-phase relative to the bulk orientation^41^. The authors do not observe clusters of misoriented β -phase, however one cannot suspect that the transformation occurs suddenly. Therefore, the formation of clusters of misoriented β-phases in samples used in our study must also be considered.

In light of the presented RBS/c results, the nature of the defect structures remains still unknown. In semiconductors, damage accumulation usually leads to amorphization^14,42^, which is a controversial issue for β-Ga_2_O_3_, because the theory predicts a high radiation resistance of this material. In fact, very high radiation tolerance of the γ-Ga_2_O_3_ only has been shown to date^28^. The RBS/c studies are not conclusive in this regard. The saturation of the RDA-type defect at the random level could be explained by amorphization or the other stable defect structures, which are visible via the RBS/c technique as channel blocking defects. For this reason, the supporting high resolution TEM and SEM studies were also performed.

**Figure 7:**
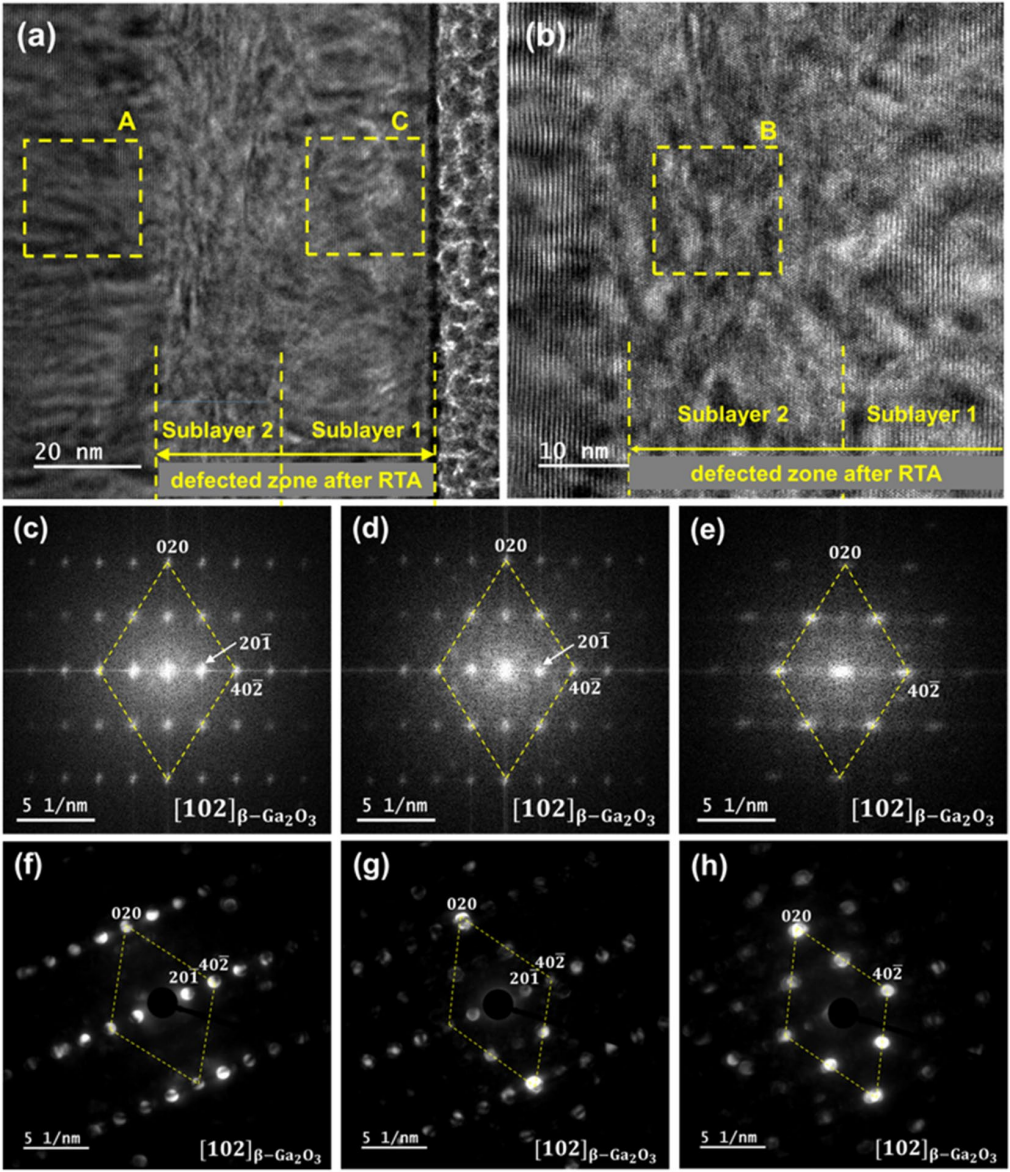
HRTEM image of β-Ga_2_O_3_ implanted with 1 × 10^15^ Yb ions/cm^2^ and annealed in O_2_ at 800 °C for 10 min (**a**), magnified HRTEM image of the sublayers (**b**) with the corresponding FFT(**c**-**d**), acquired from the areas A-C, respectively, and NBD (**f**-**h**) patterns.

**Figure 8:**
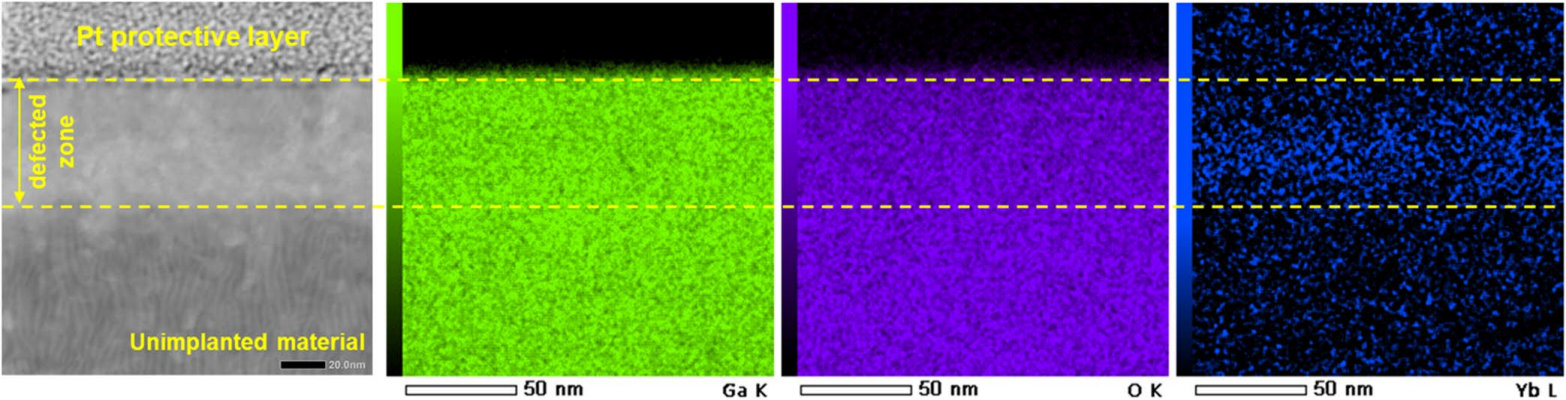
EDS maps of concentration of Ga, O, and Yb ions in (-201) oriented β-Ga_2_O_3_ single crystals implanted with 1 × 10^15^ Yb ions/cm^2^ and annealed in O_2_ at 800^o^C for 10 min.

In the TEM images presented in Fig. 7 obtained for an annealed sample of (− 201) β-Ga_2_O_3_ implanted with a Yb ion fluence of 1 × 10^15^ /cm^2^, two damage zones are still clearly visible. As depicted in Fig. 7a and b sublayer 1 is located near the surface (0–30 nm), while sublayer 2, is around 20 nm thick, and corresponds to the region of elevated Yb ions concentration (30–50 nm) (see Fig. 8). The values of these sublayer thicknesses after annealing are consistent with the RBS/c results. It is worth recalling that the defective layer directly after implantation (before annealing) reached 80 nm, and its thickness was reduced to 50 nm after annealing. As can be seen on Fast Fourier Transform (FFT) patterns as well as Nano-Beam Electron Diffraction (NBD) patterns all the marked areas were identified as the monoclinic β-Ga_2_O_3_ phase with the [102] zone axis. The well-defined atomic structure of the β-Ga_2_O_3_ near the surface suggests good recovery of the material’s initial structure during the annealing process and confirms the reduction of the γ-phase in the deeper damage zone. Moreover, the FFT/NBD observations do not show the presence of Yb_2_O_3_ clusters . However, the patterns taken from different areas reveal some differences. Looking at the FFT pattern, captured from near the surface region (area C), shown in Fig. 7d, and the corresponding NBD pattern shown in Fig. 7g it can be noticed that they are very similar to the patterns shown in Fig. 7c, and f captured from area A located near the interface, where the deeper RDA-type defect assigned to the γ-phase existed before annealing. In contrast, some variations are observed in the FFT/NBDs obtained for sublayer 2, captured from area B (see Fig. 7e, h), compared to areas A and C. Although the crystal structure of sublayer 2 can also be described as β-Ga_2_O_3_, the differences are due to the absence of the (20̅1) reflection, which is visible in the FFT/NBDs for both areas: A and C. In the perfect crystal structure of β-Ga_2_O_3_, this reflection is forbidden. However, it is typically observed in SAED patterns as a result of structural modulations caused by the occurrence of agglomerates of oxygen vacancies (Vo) in the material structure ^43^. The absence of the (20$\:\stackrel{-}{1}$) reflections in the case of sublayer 2 may indicate the lack of such agglomerates in this area. The (20$\:\stackrel{-}{1}$) reflection seems to be weaker from sublayer 1 (area C), which allows the logical assumption that the amount of Vo decreases with annealing in an oxygen atmosphere due to the diffusion of oxygen atoms in the near-surface region.

At this point, we cannot prove the existence of Vo clusters or explain why they form. However, the assumption of the truth of this hypothesis clues about the movement of atoms within the implanted layer, which can lead to stoichiometric perturbations in the corresponding sublayers. This, in turn, it could explain the observed saturation at the random level of the RBS-aligned spectrum near the surface.

## Conclusions

We have studied the ion-induced defect structure and the Yb behavior in the (010) and (-201)-oriented β-Ga_2_O_3_, as well as the thermal annealing effect for both studied crystals orientations. Our results demonstrate that the kinematics of the defects accumulation process is quite similar for both oriented crystals. However, significant differences in the number of extended defects are observed, with a much lower level of them in the (010)-oriented β-Ga_2_O_3_ compared to the (-201) samples. In contrast to many previous works, the results presented here evidently indicate that two different RDA types of defects develop in the implanted zone when Yb ion fluence exceeds 1 × 10^13^ /cm^2^. According to our understanding, these defects initially coexist as domains of different phases rather than layers. Increasing ion fluence and the accumulation of the stress lead to a defect structure transformation. This phenomenon is the driving force behind the migration or pushing of one of the RDA types of defects towards the deeper depth. It takes place for the Yb ion fluence of about 1 × 10^14^ /cm^2^ (~ 0.6 dpa), and after that, the phases develop in depth-separated damage zones. We have found that the damage zone near the surface is an amorphous phase, while the deeper damage zone is associated with the crystalline-to-crystalline β- γ phase transitions. Both radiation-induced phases disappear after the annealing. The nature of the defects in both zones of the recovered β-Ga_2_O_3_ after annealing remains unknown. However, an increase in the number or size of extended defects in this area was clearly noticed. In light of the presented results, the disturbance of the stoichiometry can be suspected, as well as the formation of clusters of misoriented β-phases in our samples needs to be considered. Additionally, our research shows that Yb ion lattice sites in both (010) as well as (-201)-oriented β-Ga_2_O_3_ are mainly of the interstitial type and do not change after the RTA treatment. Notably, after the RT implantation or 10 min. RTA at 800 °C in an oxygen atmosphere, no Yb ion diffusion towards the surface was observed. In our opinion, the present results make a significant step forward in the understanding of the radiation sensitivity of β-Ga_2_O_3_ and the annealing effect. However, further characterization methods are necessary for the understanding of the nature of the defects.

## Methods

(-201) and (010)-oriented β-Ga_2_O_3_ commercial single crystals manufactured by Tamura Corporation, Japan, were used in the studies. Both differently oriented crystals were n-type, initially undoped (UID) with the electron concentration level ≤ 9 × 10^17^ /cm^3^. The samples were 15 × 10 mm in size and 0.68 mm thick. The selected ion implantation energy was equal to 150 keV. Ion implantation was performed at Helmholtz-Zentrum Dresden-Rossendorf, Germany (HZDR) at room temperature (RT) and with a tilt of ~ 7° to avoid channeled implantation. On each of the samples, four differently damaged areas were created by ion implantation through the use of a mask: virgin and three fields implanted with Yb ions up to the fluences of 1 × 10^13^, 1 × 10^14^, and 1 × 10^15^ /cm^2^. These fluences were chosen based on our previous studies of the damage accumulation process in Yb-bombarded β-Ga_2_O_3_ as they cause damage near the defect transformation thresholds^30^. Four differently damaged fields on the samples were examined by the standard RBS/c measurements, with 1.7 MeV He^+^ ions using the Van de Graaff accelerator at HZDR. In the RBS/c experiments, the silicon detector at a scattering angle of 170° was used, with a depth resolution < 5 nm and an energy resolution < 20 keV. Subsequently, the implanted samples were exposed to high temperatures through rapid thermal annealing (RTA). RTA was performed at 800 °C, in an oxygen atmosphere for 10 min using an Accu Thermo AW-610 from Allwin21 Corporation system at the Institute of Physics, Polish Academy of Sciences (PAS). It has been found that such conditions of annealing provide effective lattice recovery and the most intense emission of RE ions in β-Ga_2_O_3_^18^. Finally, the RBS/c experiments were repeated on the annealed samples. The collected RBS/c spectra, obtained for the implanted and implanted-annealed samples were subjected to qualitative and quantitative analyses using a Monte Carlo simulation code, called *McChasy-1*^32,33^. The unique property of the *McChasy-1* program is its ability to distinguish the contributions to the RBS-*aligned* spectra (i.e., those obtained in the channeling mode) that originated from both simple and extended defects. The transmission electron microscopy (TEM) observations were also performed using a JEOL JEM-F200 microscope operating at 200 kV. Thin samples for the TEM studies were prepared using a focused ion beam (FIB) lift-out method with the application of a ThermoFisher Scientific Helios 5 UX dual-beam scanning electron microscope (SEM). To avoid the formation of Ga^+^-induced structural defects, the final polishing was performed with the accelerated voltage of 2 kV.

## Electronic supplementary material

Below is the link to the electronic supplementary material.

Supplementary Material 1.

## Data Availability

The datasets generated and analyzed during the current study are available from the corresponding author upon reasonable request.

## References

[1] Higashiwaki, M., Sasaki, K., Kuramata, A., Masui, T. & Yamakoshi, S. *Gallium oxide (Ga2O3) metal-semiconductor field-effect transistors on single-crystal β-Ga2O3 (010) substrates*, Appl. *Phys. Lett.* **100**, 013504. 10.1063/1.3674287 (2012).

[2] Green, A. J. et al. *β -Ga*_*2*_*O*_*3*_*MOSFETs for Radio Frequency Operation*. *IEEE Electron. Device Lett.* **38**, 790. 10.1109/LED.2017.2694805 (2017).

[3] Stepanov, S. I., Nikolaev, V. I., Bourgov, V. E. & Romanov, A. E. Gallium oxide: Properties and applications - a review. *Rev. Adv. Mater. Sci.* **44**, 63 (2016).

[4] Pearton S.J. et al. A review of Ga2O3 materials. *Appl. Phys. Rev.* **5**, 011301. 10.1063/1.5006941 (2018).

[5] Chikoidze, E. et al. *Mater. Today Phy* **3**, 118 (2017).

[6] Miyata, T., Nakatani & Minami, T. Gallium oxide as host material for multicolor emitting phosphors. *J. Lumin.* **87–89**, 1183 (2000).

[7] Monroy, E., Omne`s, F. & Calle, F. Wide-bandgap semiconductor ultraviolet photodetectors. *Semicond. Sci. Technol.* **18**, R33. 10.1088/0268-1242/18/4/201 (2003).

[8] Binet, L. & Gourier, J. Origin of the blue luminescence of β-Ga2O3. *J. Phys. Chem. Solids*. **59**, 1241 (1998).

[9] Onuma, T. et al. Modeling and interpretation of UV and blue luminescence intensity in β-Ga2O3 by silicon and nitrogen doping. *Appl. Phys.* **124**, 075103 (2018).

[10] Vasyltsiv, V. et al. Luminescence and conductivity of β-Ga2O3 and β-Ga2O3:mg single crystals. *Acta Phys. Pol., a*. **141**, 312–318 (2022).

[11] Favennec, P. N., L’Haridon, H., Moutonnet, D., Salvi, M. & Gauneau, M. *Rare Earth Doped Semiconductors*, in: Pomrenke, Klein, Langer (Eds.), MRS Symposia Proceedings, Materials Research Society, Pittsburgh, PA, 31, 181 (1993).

[12] Santos, N. F. et al. Luminescence studies on SnO2 and SnO2:Eu nanocrystals grown by laser assisted flow deposition. *Appl. Surf. Sci.* **258**, 9157–9161. 10.1039/c4cp06114d (2012). 10.1039/c4cp06114d 25932704

[13] Nogales, E. et al. *Visible and infrared luminescence study of Er doped β-Ga*_*2*_*O*_*3*_*and Er*_*3*_*Ga*_*5*_*O*_*12*_. *J. Phys. D: Appl. Phys.* **41**, 065406. 10.1088/0022-3727/41/6/065406 (2008).

[14] Lorenz, K. et al. *Oxide-based Materials and Devices V*, edited by Ferechteh H. Teherani, David C. Look, David J. Rogers, Proc. of SPIE Vol. 89870 M (2014). (1987).

[15] Williams, J. S. Ion implantation of semiconductors. *Mater. Sci. Eng.* **A253**, 8–15 (1998).

[16] Ratajczak, R. et al. Luminescence in the visible region from Annealed Thin ALD-ZnO films implanted with different Rare Earth ions. *Phys. Status Solidi A*. **215**, 1700889 (2018).

[17] Zhou, X. et al. Interplay of defect levels and rare earth emission centers in multimode luminescent phosphors. *Nat. Commun.* **13**, 7589 (2022). 36481731 10.1038/s41467-022-35366-3 PMC9732309

[18] Sarwar, M. et al. Crystal lattice recovery and optical activation of Yb implanted into the beta-Ga2O3 crystal. *Materials*. **17**, 39791 (2024). 10.3390/ma17163979 PMC11356592 39203157

[19] López, I. et al. Study of the relationship between crystal structure and luminescence in rare-earth-implanted Ga2O3 nanowires during annealing treatments. *J. Mater. Sci.* **49**, 1279–1285 (2014).

[20] Lorenz, K. et al. *Doping of Ga2O3 bulk crystals and NWs by ion implantation*, Proc. of SPIE Vol. 8987 89870 M-1 (2014).

[21] Wendler, E., Treiber, E., Baldauf, J., Wolf, S. & Ronning, C. *High-level damage saturation below amorphization in ion implanted β-Ga*_*2*_*O*_*3*_. *Phys. Res. B*. **379**, 85–89 (2016). Nuclear Instruments and Methods in.

[22] Azarov, A. et al. Interplay of the disorder and strain in gallium oxide. *Sci. Rep.* **12**, 15366 (2022). 36100627 10.1038/s41598-022-19191-8 PMC9470558

[23] Azarov, A., Venkatachalapathy, V., Monakhov, E. V. & Kuznetsov, A. Y. Dominating migration barrier for intrinsic defects in gallium oxide: dose-rate effect measurements. *Appl. Phys. Lett.* **118**, 232101 (2021).

[24] Anber, E. A. et al. *Structural transition and recovery of Ge implanted β-Ga*_*2*_*O*_*3*_. *Appl. Phys. Lett.* **117**, 152101 (2020).

[25] Polyakov, A. Y. et al. *Conducting surface layers formed by hydrogenation of O-implanted β-Ga*_*2*_*O*_*3*_. *J. Alloys Compd.* **945**, 169258 (2023).

[26] Karjalainen, A. et al. *Phys. Rev. B* **102**, 195207 (2020).

[27] Kjeldby, S. B. et al. Radiation-induced defect accumulation and annealing in Si-implanted gallium oxide. *J. Appl. Phys.* **131**, 125701 (2022).

[28] García-Fernández, J. et al. *Formation of c-Ga*_*2*_*O*_*3*_*by ion implantation: Polymorphic phase transformation of β-Ga2O3*. *Appl. Phys. Lett.* **121**, 191601 (2022).

[29] Azarov, A. et al. Kuznetsov, Univ*ersal radiation tolerant semiconductor*. *Nat. Commun.* **14**, 4855. 10.1038/s41467-023-40588-0 (2023). 37563159 10.1038/s41467-023-40588-0 PMC10415340

[30] Sarwar, M. et al. Defect Accumulation in β-Ga2O3 implanted with yb. *Acta Mater.* **268**, 119760. 10.1016/j.actamat.2024.119760 (2024).

[31] Azarov, A. et al. Disorder-Induced Ordering in Gallium Oxide Polymorphs. *Phys. Rev. Lett.* **128**, 015704. 10.1103/PhysRevLett.128.015704 (2022). 35061456 10.1103/PhysRevLett.128.015704

[32] Jozwik, P. et al. *Advanced Monte Carlo Simulations for Ion-Channeling Studies of Complex Defects in Crystals*, in *Theory and Simulation in Physics for Materials Applications: Cutting-Edge Techniques in Theoretical and Computational Materials Science*, edited by E. V. Levchenko, Y. J. Dappe, and G. Ori (Springer International Publishing, Cham, 133–160, (2020).

[33] Jóźwik, P., Caçador, A., Lorenz, K., Ratajczak, R. & Mieszczyński, C. Monte Carlo Simulations of Ion Channeling in the Presence of dislocation loops: New Development in the McChasy Code. *Phys. Res. Sect. B: Beam Interact. Mater. Atoms*. **538**, 198 (2023). Nuclear Instruments and Methods in.

[34] Fu, H. et al. A comparative study on the Electrical properties of Vertical (201) and (010) β-Ga₂O₃ Schottky Barrier diodes on EFG single-crystal substrates. *IEEE Trans. Electron. Devices*. 1–7. 10.1109/TED.2018.2841904 (2018).

[35] Chu, W. K., Mayer, J. W. & Nicolet, M-A. *Backscattering Spectrometry*. Academic Press, New York 225 (1978).

[36] Ziegler, J. F. *SRIM-* Nucl. Instrum. Method. Phys. Res. B 1027–1036 (2004). (2003).

[37] Huang, H-L. et al. Hwang; *atomic scale defect formation and phase transformation in Si implanted β-Ga2O3*. *APL Mater.* **11** (6), 061113. 10.1063/5.0134467 (2023).

[38] Demchenko, I. N., Ratajczak, R., Melikhov, Y., Konstantynov, P. & Guziewicz, E. Valence band of ZnO: yb probed by resonant photoemission spectroscopy. *Mater. Sci. Semiconduct. Process.* **91**, 306–309 (2019).

[39] Yoo, T. et al. Atomic-scale characterization of structural damage and recovery in Sn ion-implanted β-Ga2O3. *Appl. Phys. Lett.* **121** (7), 072111. 10.1063/5.0099915 (2022).

[40] García-Fernández, J. et al. In situ atomic-resolution study of transformations in double polymorph γ/β-Ga2O3 structures. *Mater. Adv.*10.1039/D3MA01011B (2024).

[41] Azarov, A. et al. *Optical activity and phase transformations in γ/β Ga*_*2*_*O*_*3*_*bilayers under annealing*. *Adv. Opt. Mater.* 2401325. 10.1002/adom.202401325 (2024).

[42] Wendler, E. Mechanisms of damage formation in semiconductors. *Nucl. Instrum. Methods Phys. Res. B*. **267**, 2680–2689 (2009).

[43] Víllora, E. G. et al. *Electron microscopy studies of microstructures in β-Ga*_*2*_*O*_*3*_*single crystals*. *Mater. Res. Bull.* **37** (4), 769–774. 10.1016/S0025-5408(02)00689-X (2002).

