# Supplementary material for: Anisotropy of radiation-induced defects in Yb-implanted β-Ga2O3
Source: Sci Rep. 2024 Oct 22;14:24800. doi: 10.1038/s41598-024-75187-6 (PMC11494172; doi:10.1038/s41598-024-75187-6)
Supplement: Supplementary file 1 — Supplementary Material 1. [file 41598_2024_75187_MOESM1_ESM.pdf]

Supplementary materials

Anisotropy of radiation-induced defects in Yb-implanted β-Ga_2O_3

R. Ratajczak, M. Sarwar, D. Kalita, P. Jozwik, C. Mieszczynski, J. Matulewicz, M. Wilczopolska, W. Wozniak, U. Kentsch, R. Heller and E. Guziewicz

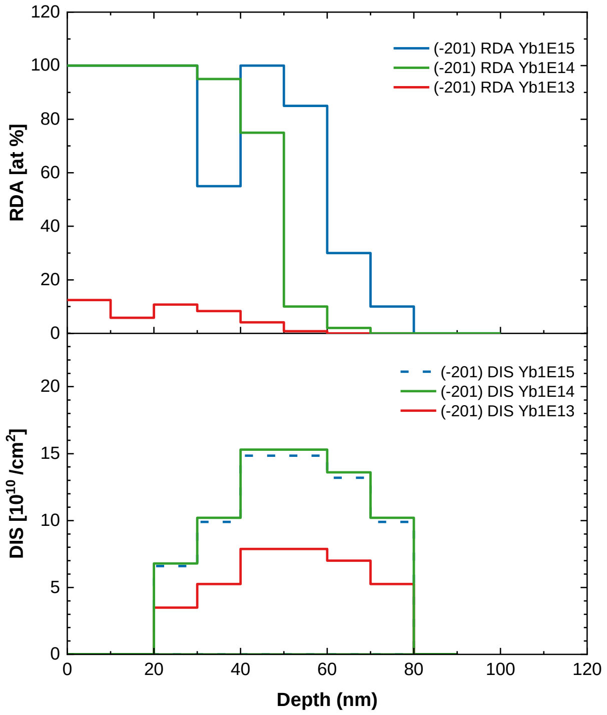

Figure 1 SM. Distribution of simple (RDA) and extended (DIS) defects obtained by McChasy simulations for (-201) oriented β-Ga_2O_3 implanted with Yb-ions of different fluences.

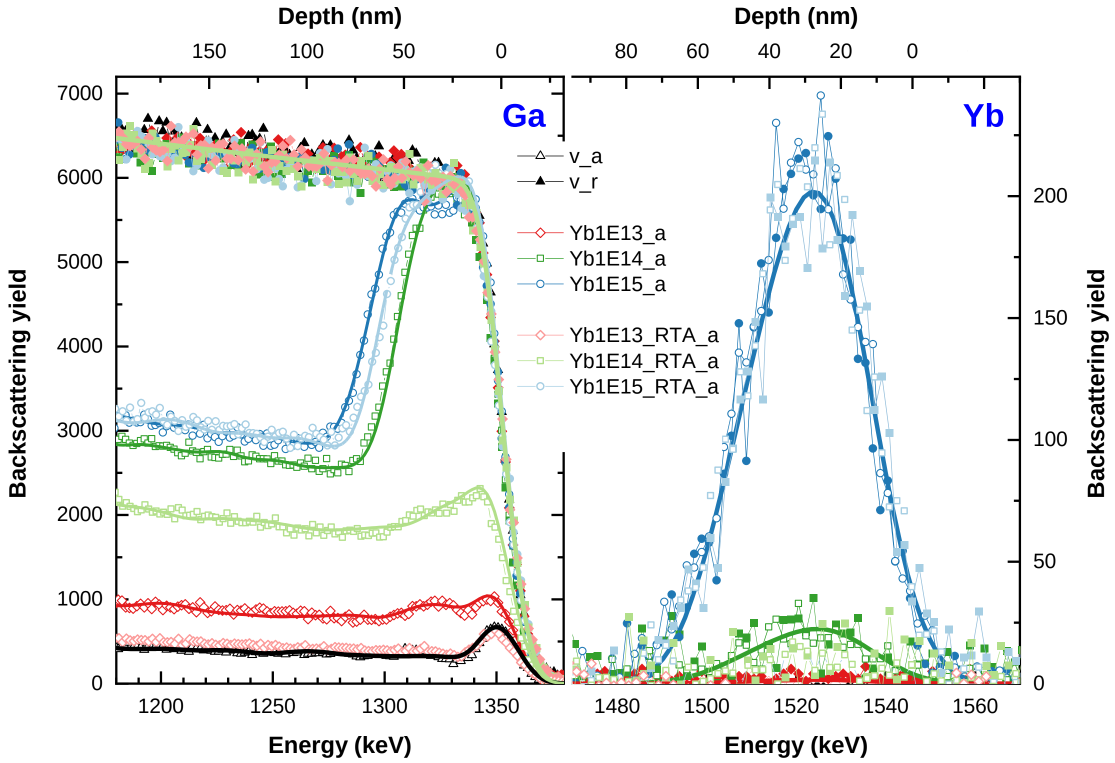

Figure 2 SM. Random (solid symbols) and aligned (open symbols) RBS experimental spectra obtained for (-201) oriented β-Ga_2O_3 single crystals implanted with different fluences of Yb ions and annealed in O_2 at 800°C for 10min.

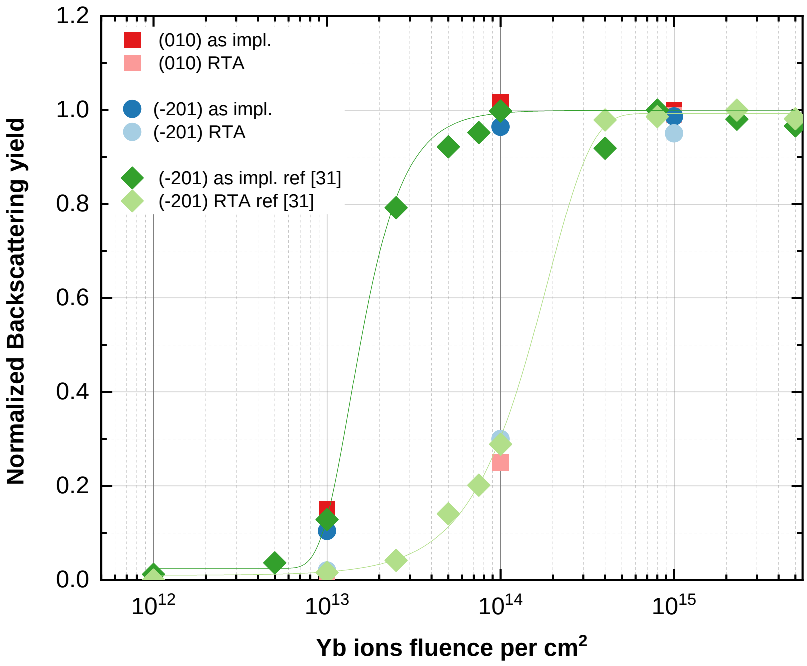

Figure 3 SM. Dependence of the backscattering yield (normalized to the random level) in the damage zone for (-201) and (010) oriented β-Ga_2O_3 implanted with Yb and post-implantation annealed for 10 min at 800 °C in O_2, measured for the aligned spectra at the energy region of 1325-1352 keV and normalized to the random level.

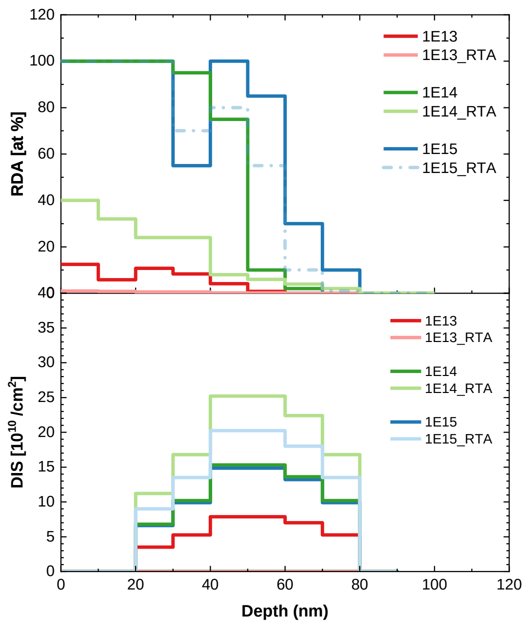

Figure 4 SM. Distribution of simple (RDA) and extended (DIS) defects obtained by McChasy simulations for (-201) oriented β-Ga_2O_3 single crystals implanted with different fluences of Yb ions and annealed in O_2 at 800°C for 10min
